# Clustering of plaques contributes to plaque growth in a mouse model of Alzheimer’s disease

**DOI:** 10.1007/s00401-013-1137-2

**Published:** 2013-06-18

**Authors:** Joanna F. McCarter, Sabine Liebscher, Teresa Bachhuber, Claudia Abou-Ajram, Mark Hübener, Bradley T. Hyman, Christian Haass, Melanie Meyer-Luehmann

**Affiliations:** 1 Adolf Butenandt Institute, Biochemistry, Ludwig-Maximilians-University, Munich, Germany; 2 Department Synapses-Circuits-Plasticity, Max Planck Institute of Neurobiology, 82152 Martinsried, Germany; 3 Massachusetts General Institute for Neurodegenerative Diseases, MGH/Harvard Medical School, Charlestown, MA USA; 4 German Center for Neurodegenerative Diseases (DZNE), 80336 Munich, Germany; 5 Munich Cluster for Systems Neurology (SyNergy), Munich, Germany; 6 Neurocenter, Department of Neurology, Albert-Ludwigs-University of Freiburg, Breisacher Str.64, 79106 Freiburg, Germany

**Keywords:** Alzheimer’s disease, Amyloid plaques, APPPS1 transgenic mice, Two-photon in vivo imaging

## Abstract

Amyloid-β (Aβ) plaque deposition plays a central role in the pathogenesis of Alzheimer’s disease (AD). Post-mortem analysis of plaque development in mouse models of AD revealed that plaques are initially small, but then increase in size and become more numerous with age. There is evidence that plaques can grow uniformly over time; however, a complementary hypothesis of plaque development is that small plaques cluster and grow together thereby forming larger plaques. To investigate the latter hypothesis, we studied plaque formation in APPPS1 mice using in vivo two-photon microscopy and immunohistochemical analysis. We used sequential pre- and post-mortem staining techniques to label plaques at different stages of development and to detect newly emerged plaques. Post-mortem analysis revealed that a subset (22 %) of newly formed plaques appeared very close (<40 μm) to pre-existing plaques and that many close plaques (25 %) that were initially separate merged over time to form one single large plaque. Our results suggest that small plaques can cluster together, thus forming larger plaques as a complementary mechanism to simple uniform plaque growth from a single initial plaque. This study deepens our understanding of Aβ deposition and demonstrates that there are multiple mechanisms at play in plaque development.

**Electronic supplementary material:**

The online version of this article (doi:10.1007/s00401-013-1137-2) contains supplementary material, which is available to authorized users.

## Introduction

Amyloid-β (Aβ) plaques are a major pathological hallmark of AD. Although plaque deposition is not thought to correlate with disease severity [12], there are numerous studies proving that the microenvironment around plaques is affected. For example, there is considerable evidence for altered neuronal structure and function in AD, such as the occurrence of hyperactive neurons, neuritic dystrophies and the loss of synapses which are almost exclusively observed within the peri-plaque region [5, 8, 18, 19, 23, 28, 31]. These data emphasize the potential pathological importance of plaques for the disease and that plaques do not have a mere epiphenomenal role and, therefore, warrant further scientific investigation.

Previous post-mortem analyses of brain sections from an AD mouse model at different time points showed that plaques become larger on average over time with increasing pathology progression [27]. Post-mortem analyses of brain sections from AD patients revealed variable plaque size in individuals. No change in plaque size occurred with increasing duration of disease, consistent with processes that halt plaque growth over time. Interestingly, a strong correlation between earlier age of disease onset and larger plaque size distribution was noted, suggesting that individual factors that impact rapidity or frequency of plaque deposition might also impact their ultimate size [29].

Our current study reexamines the question of how these large plaques form. In vivo two-photon imaging revealed that plaques can develop rapidly de novo within 24 h and remain relatively small and at a stable size for at least 2 subsequent weeks [24]. Long-term in vivo imaging studies of plaques over several months demonstrated a gradual uniform growth of amyloid-β plaques over time [7, 13]. These studies imply that initial plaque formation is very fast, followed by a gradual growth phase of individual plaques. An alternative model proposed that random spatial allocation was not sufficient to match the pattern of plaque distribution found in post-mortem histological tissue, but rather that plaques favorably emerged in the vicinity of an already existing plaque [32]. Inspired by these findings, we examined whether one mechanism that might lead to large plaques could be the cluster formation of pre-deposited and newly emerged plaques rather than uniform growth of a single, initial plaque.

In this study, we labeled plaques at multiple stages of development to test the hypothesis that new plaques preferentially deposit in clusters around pre-existing plaques and to evaluate the occurrence of large plaques formed from multiple precursors. Our data provide the first direct in vivo evidence of plaque growth resulting from consolidation of clusters of multiple plaques.

## Materials and methods

### Animals

For this study, we used male heterozygous APPPS1 transgenic mice [27], expressing human APP_KM670/671NL_ and PS1_L166P_ under the control of the Thy-1 promoter [2]. Mice were group housed under specific pathogen-free conditions. Animals had access to food and water ad libitum and were kept under a 12/12 h light–dark cycle. All animal procedures were carried out in accordance with an animal protocol approved by the government of Upper Bavaria.

### Pre-mortem staining of amyloid plaques

For pre-mortem plaque labeling, we chose Methoxy-XO4 (Neuroptix, Acton, MA, USA), a congo-red derivative, which has been shown to readily cross the blood brain barrier [16], bind to dense core plaques and importantly, remain bound to them for a period of at least 90 days [9]. Methoxy-XO4 was administered intraperitoneally (i.p.) at a concentration of 3.33 mg/kg (3.3 % volume of 10 mg/ml stock solution in DMSO (light shielded), 6.66 % volume Cremophore EL (Sigma Aldrich, St. Louis, MO, USA) in 90 % volume phosphate buffered saline (PBS)) [16] to the mice to label the cerebral amyloid plaques present at day 0. After different post-injection intervals of 1 day, 1 month or 4 months, we performed either acute in vivo two-photon imaging or post-mortem analysis. Animals were all 3 months old at the time of Methoxy-XO4 injection, apart from one group of animals (*n* = 4) that was injected at 2 months of age followed by a post-injection time of 2 weeks. A complete list with the number of animals used for each experiment is listed in Suppl. Table 1.

### Cranial window surgery and two-photon in vivo imaging

The mice were anaesthetized with an i.p. injection of a mixture of Ketamine (10 mg/kg) and Xylazine (20 mg/kg). Under semi-sterile conditions, a cranial window was implanted as previously described [14, 31]. Briefly, a 6 mm diameter circle of cranial bone was removed using a micro drill (Stoelting, Wood dale, IL, USA) over the parietal cortex and the dura mater detached [30]. 20 μl of anti-Aβ antibody 6E10 (Sigma Aldrich, St. Louis, MO, USA) covalently labeled with Alexa 594 (antibody labeling kit, InvivoGen, San Diago, CA, USA) was topically applied to the surface of the brain for 30 min followed by two 30 min washes with sterile PBS. An 8 mm round glass cover slip was mounted over the exposed brain using Pattex Ultra gel (Henkel, Duesseldorf, Germany) and Paladur dental cement (Heraeus, South Bend, IN, USA) to close the craniotomy, with Paladur built up around the window to provide a well for the water immersion objective. Acute two-photon imaging was carried out immediately using Olympus FV1000 with Mai Tai Deep See Laser (Spectra Physics, Newport Corporation, Franklin, MA, USA) with an excitation wavelength of 850 nm. Emission filters of 420–500 nm (Methoxy-XO4) and 590–650 nm (Alexa 594) were used. To visualize the deposition of new plaques, we used an Olympus 20× water immersion objective to acquire image stacks of 634 × 634 × 150 μm (xyz dimensions) with 5 μm z-increments and 512 × 512 pixel resolution. Cortical tissue immediately underneath the surface was not included in the analysis due to potential contamination of non-amyloidogenic autofluorescence signals derived from blood or bone marrow cells. Following acute imaging, mice were killed via cervical dislocation and the brains processed for post-mortem analysis. For chronic in vivo imaging, APPPS1/GFP transgenic mice at the age of 2.5 months were implanted with a cranial window as described above, without application of the antibody and the dura mater left intact. After a recovery period of 4 weeks, successive Aβ plaque imaging of the same regions was carried out weekly for 1 month. Mice were re-imaged after 4 and 5.5 months. Methoxy-XO4 (3.3 mg/kg) was administered i.p. 24 h prior to each imaging session.

### Post-mortem analysis

Brains were removed from the skull and fixed in 4 % paraformaldehyde (Roth Roti^®^-Histofix, Karlsruhe, Germany) for 48 h, followed by incubation in 30 % sucrose (in PBS, pH 7.5) for a further 48 h. The brains were frozen and 25 μm thick coronal frozen sections were cut with a sliding microtome (SM200R, Leica Biosystems, Wetzlar, Germany). Immunofluorescent staining was performed on every tenth section with the anti-Aβ antibody 3552 ([25]; 1:3,000, overnight at 4 °C; anti-rabbit secondary antibodies (Invitrogen, Life Technologies, Paisley, UK): Alexa555, 1:1,000 1 h at room temperature or Alexa488, 1:250, 1 h at room temperature) or anti-Aβ antibody 4G8 (Covance, 1:150, overnight at 4 °C; anti-mouse secondary antibody Alexa488 (Invitrogen), 1:250, 1 h at room temperature). Omission of primary antibodies showed no detectable staining. Alternatively, dense core plaques were stained with Thiazin Red (Sigma Aldrich, 2 μM solution in PBS) for 20 min at room temperature followed by 3 × 5 min PBS washes. Post-mortem Methoxy-XO4 staining was carried out following imaging of pre-mortem Methoxy-XO4 labeling: the cover slip was removed and the mounted sections washed in PBS and incubated in a solution containing 100 μM Methoxy-XO4 dissolved in 40 % ethanol (adjusted to pH 10) for 10 min. The slide was then briefly dipped in water five times, put in 0.2 % NaOH in 80 % ethanol for 2 min and washed again in water for 10 min. Sections were mounted on object slides using Aqua-Poly-mount mounting media (PolySciences, Eppelheim, Germany). Post-mortem multichannel images were taken using an Olympus BX61 (Olympus, Tokyo, Japan) fluorescent microscope with an EXFO-Xcite laser. A 20× UPLSAPO air objective and the following filters were used: U-MNUA (for Methoxy-XO4 signal, excitation at 345 nm, emission at 455 nm), Cy3 (for the secondary antibody Alexa 555 and Thiazin Red, excitation at 550 nm, emission at 565 nm) and U-MNIBA (for Alexa 488, excitation at 495 nm, emission at 519 nm). Images (688 μm × 512 μm) were taken with an F-view 11FW camera (Olympus) over dorsal, lateral and ventral cortical regions. Six regions of interest (ROIs) were imaged per brain section, resulting in a total of 89 ± 10.3 (mean ± standard deviation) ROIs per animal.

### Image processing and data analysis

Image analysis was done using Photoshop CS5 (Adobe Systems Inc., San Jose, CA, USA). Background signal was manually subtracted using the linear image histogram tool. A ‘new’ plaque was defined as a structure being Aβ antibody positive but Methoxy-XO4 negative. The distance between plaques was measured between the centers of two plaques using ImageJ software (National Institutes of Health freeware). Plaques with a core to core distance of less than 40 μm were categorized as ‘in the vicinity’ of each other and if multiple (more than 1) new plaques were in the vicinity of a pre-existing plaque, then these were denoted as ‘flower plaques’ due to their appearance as petals around a central core. To determine the size distribution of plaques, we analyzed images after smoothing, thresholding and background subtraction steps in ImageJ. Aβ antibody fluorescence ≥30 pixels (≥5.5 μm^2^) was counted as plaques. Autofluorescent signal from particles of section edges were manually removed from the data set. Multicore plaques were classified as plaques bearing 2 or more, clearly separate, Methoxy-XO4 positive cores that were located within the boundaries of a single Aβ antibody labeled plaque. To determine the chance level of plaque deposition in close vicinity of a pre-existing plaque, we computer-generated new plaques in random locations using a custom-written program in MATLAB (Mathworks). A plaque-free region was chosen to estimate the level of the background noise. Detection stringency was set to nine times the standard deviation of the background noise and putative plaques had to consist of a minimum area of 20 pixels (5 μm^2^). All imagery was visually checked for correct plaque detection. For the simulation, the same number of newly deposited plaques that were counted in the original image was placed at random locations within the image, and their distances to the center of the closest pre-existing plaque determined. For each image, 1,000 iterations of the simulation were conducted, and the proportion of ‘new in vicinity’ plaques occurring by chance was compared to the original proportion of new plaques.

### Statistics

All data sets were tested for normality with the D’Agostino-Pearson omnibus K2 normality test with a significance level set to *p* = 0.05, before the appropriate parametric or non-parametric statistical comparison test was carried out with GraphPad Prism 5.04. (GraphPad Inc., La Jolla, CA, USA).

## Results

**Fig. 1:**
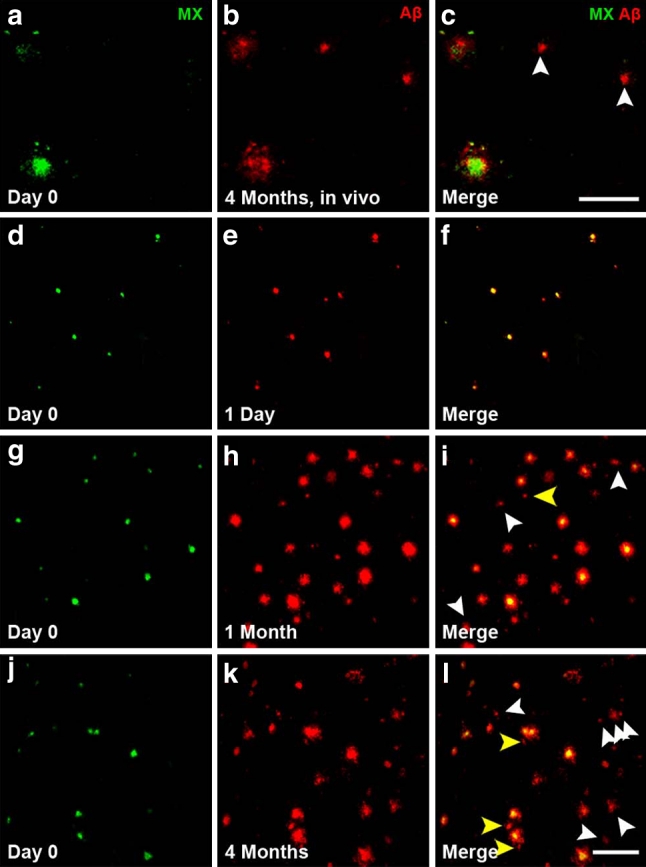
Pre-existing and new plaques revealed with dual staining technique. **a**–**c** In vivo two-photon imaging of newly appearing amyloid plaques. Methoxy-XO4 labeled pre-existing plaques in green (**a**), anti-Aβ antibody labeled plaques after 4 months post-injection as shown in red (**b**) and the merged image of the two fluorescence channels with newly developed plaques depicted with *white arrowheads* (**c**). **d**–**l** Occurrence of new plaques demonstrated by sequential pre- and post-mortem staining. Plaques that existed at day 0 (3-month-old animals) are visualized with Methoxy-XO4 (**d**, **g**, **j**). Post-mortem plaque analysis with immunohistochemical staining against Aβ (**e**) reveals almost complete co-localization at 1-day post-injection (**f**). After 1 month (**g**–**i**) and 4 months post-injection (**j**–**l**), an increasing amount of newly formed plaques (*white arrowheads*), including ‘new in vicinity’ plaques (<40 μm away from a pre-existing plaque, *yellow arrowheads*) were detectable (**i**, **l**). *MX* Methoxy-XO4. *Scale bar* **a**–**c** 50 μm, **d**–**l** 100 μm

**Fig. 2:**
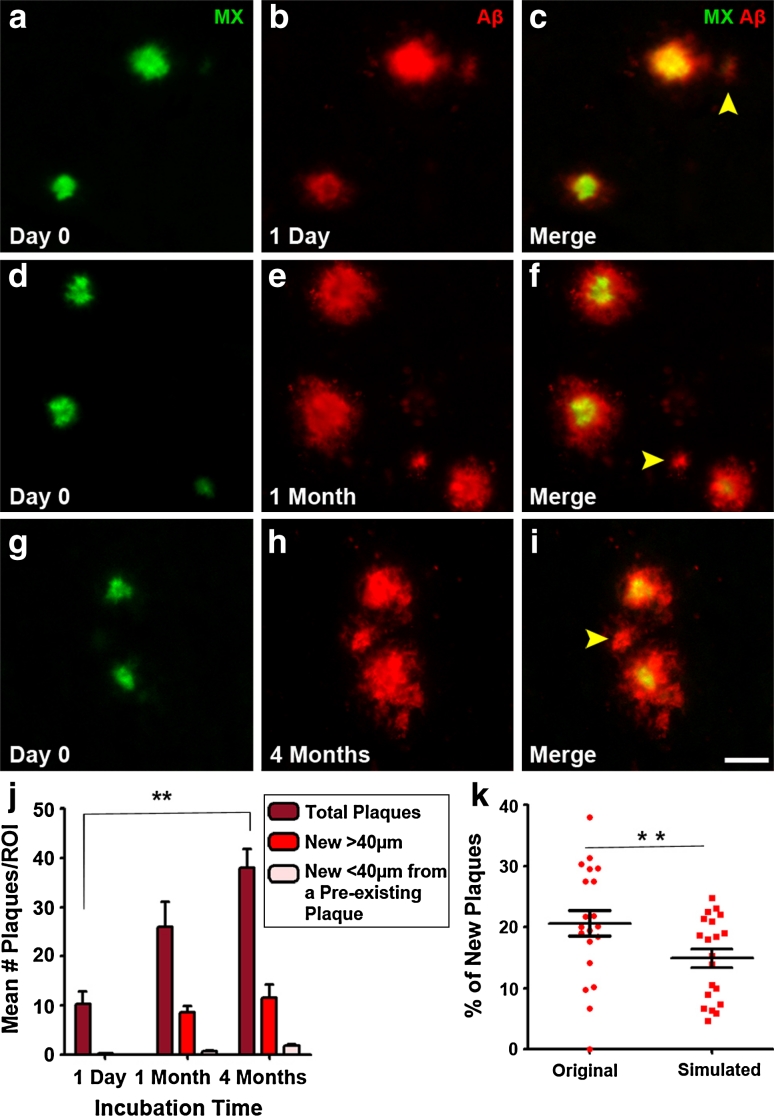
‘New in the vicinity’ plaques occur more frequently than expected by chance. (**a**–**c**) High resolution images of immunofluorescent staining 1 day, 1 and 4 months post-Methoxy-XO4 injection reveal newly developed plaques in the vicinity (<40 μm) of a pre-existing plaque (**c**, **f**, **i**, *yellow arrowheads*). Methoxy-XO4 shown in *green*, Aβ 3552 antibody staining shown in *red*. Quantification of new plaques showed an increase in both single new plaques further away (>40 μm) from pre-existing plaques and new plaques in the vicinity (<40 μm) of a pre-existing plaque over time (**j**). Mean ± SEM, *n* = 5–6 per group; Kruskal–Wallis with Dunn’s post hoc test **p < 0.01. **k** Computer-simulated random locations of new plaques revealed a significantly lower fraction of ‘new plaques in vicinity’ compared to the original data set. Each data point represents one animal. Mean ± SEM, *n* = 20; Wilcoxon matched-pairs signed rank test **p < 0.01. *Scale bar* 25 μm

We used a two-stage staining technique with different dyes and antibodies to label plaques at multiple stages of plaque development. To label plaques at ‘day 0’, the fluorescent amyloid binding dye Methoxy-XO4 was used as it is a marker of amyloid fibrils and remains bound to plaques for at least 90 days in the living mouse brain [9]. Following a post-injection time of 1 day (1 day represents the earliest possible time point for plaque analysis), 1 month or 4 months (note that as the animals were all the same age at Methoxy-XO4 injection, a longer post-injection time also corresponds to a greater age of the animals), we visualized new plaques using an antibody against Aβ (Fig. 1). With this dual staining technique, we were able to distinguish pre-existing (double labeled with Methoxy-XO4 and Aβ antibody) from newly developed (exclusively Aβ antibody stained) plaques (Fig. 1). We observed these two plaque categories with in vivo two-photon imaging, using a topically applied antibody against Aβ (Fig. 1a–c). In vivo two-photon imaging, however, only allows for the analysis of a relatively small and restricted cortical volume since antibody penetration is less than 200 μm and limited to the upper cortical layers [30]. Therefore, to gain a more comprehensive picture of new plaque appearance, we extended our study and included post-mortem brain slice analysis. Consistent with an earlier study [24], we found that 96.2 % of plaques were co-stained with Aβ antibody 1 day after Methoxy-XO4 injection with only negligible Aβ antibody positive new plaques (3.8 % ± 0.4, Fig. 1d–f). In contrast, numerous new plaques developed with longer post-injection periods (Fig. 1g–l, arrowheads). As the Aβ antibody labels diffuse Aβ in addition to the dense cores of plaques labeled by Methoxy-XO4 and may, therefore, label an additional population of plaques, we performed a post-mortem dense core stain using either Methoxy-XO4 (Suppl. Fig. 1a–c) or Thiazin Red (Suppl. Fig. 1d–f). The latter affirmed that only 0.8 % of Aβ antibody positive plaques were not dense core positive, further validating our antibody staining technique. As an additional control, we used the monoclonal antibody 4G8 as an alternative Aβ staining. This antibody was shown to label best the diffuse, fleecy amyloid in human brain sections [1]. With both antibodies (3552 and 4G8), we obtained similar results as seen by similar antibody labeled halo size after different post-injection times (compare Fig. 2c, f, i with Suppl. Fig. 2d, h, l). The population of new plaques also included plaques appearing very close (<40 μm) to pre-existing plaques we categorized as ‘new in vicinity’ (Fig. 2c, f and i, yellow arrowheads). Quantification of all plaques revealed that longer post-injection times resulted in more new plaques, including more ‘new in vicinity’ plaques (Fig. 2j). The amount of ‘new in vicinity’ plaques consistently accounted for ~22 % of all new plaques, independent of the post-injection time.

To determine if the above-mentioned 22 % of all new plaques found very close to pre-existing ones was not simply due to chance, we generated computer-simulated ‘new’ plaques in random locations and measured the distance of these randomly placed new plaques to already existing ones. Indeed, the amount of original ‘new in vicinity’ plaques was significantly higher than the random, simulated new plaques (Fig. 2k). This trend held true for all post-injection time groups, but was only significant in the short post-injection group (2-month-old animals with a 2 week post-injection time and 3 month old animals with a 1-day post-injection incubation time) (Suppl. Fig. 3 a). After 1 and 4 months post-Methoxy-XO4 injection, the data had the same trend—there were more ‘new in vicinity’ plaques in the original compared to the simulated data—however, for these groups, this difference did not reach significance (Suppl. Fig. 3b), most likely due to higher plaque density with increasing time (Suppl. Fig. 3c).

**Fig. 3:**
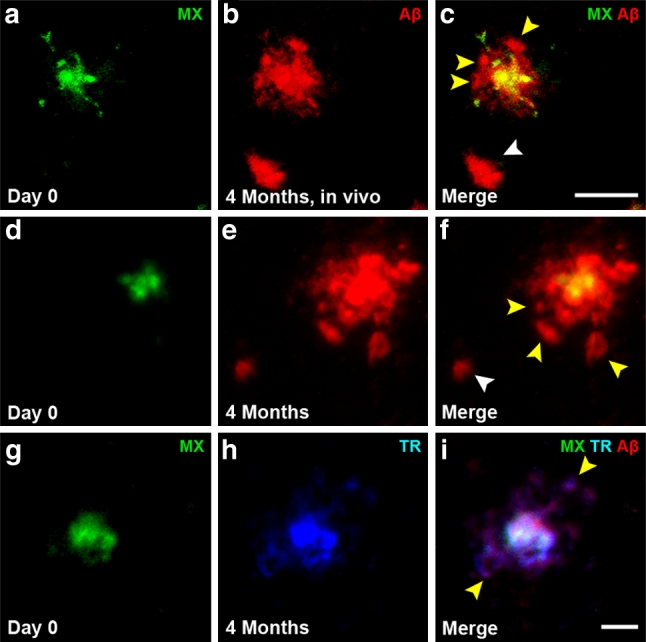
Flower plaques—new plaques cluster around pre-existing plaques (**a**–**c**). Maximum intensity projections of an in vivo image observed with a two-photon microscope 4 months post-Methoxy-XO4 injection (depicted in *green*) combined with acute topical application of fluorescently labeled Aβ antibody 6E10 staining (depicted in *red*) (**b**). The *yellow arrowheads* (**c**, **f** and **i**) indicate new plaque ‘petals’ clustering around a common pre-existing plaque creating a ‘flower plaque’. A flower plaque observed with pre-mortem Methoxy-XO4 and post-mortem Aβ antibody staining 4 months post-injection (**d**–**f**). A flower plaque visualized with post-mortem Thiazin Red 4 months post-injection (**g**–**i**) illustrating that flower plaques can also be composed of multiple dense core plaques. *Scale bar* 25 μm

**Fig. 4:**
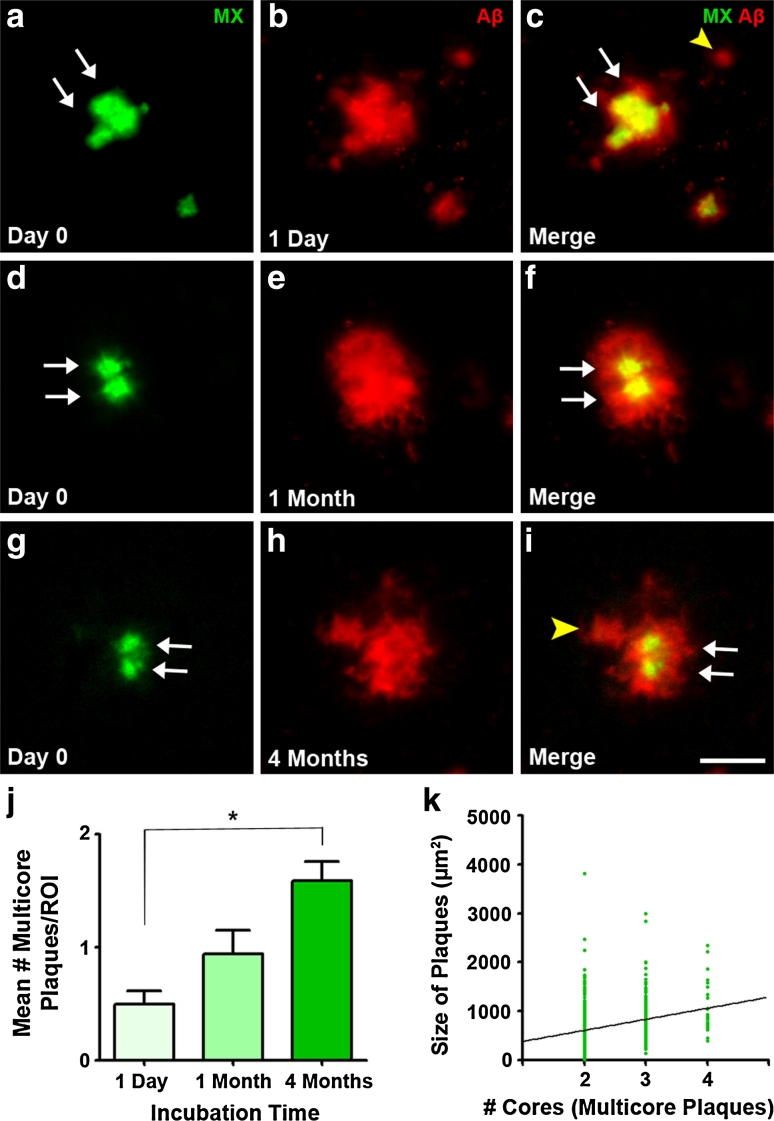
Large plaques can contain multiple dense cores at all time-points investigated. Plaques with more than one initial dense plaque core as shown by in vivo Methoxy-XO4 injection, the so-called ‘multicore plaques’, were found after 1 day (**a**–**c**), 1 month (**d**–**f**) and 4 months (**g**–**i**) post-injection. Pre-mortem Methoxy-XO4 labeling (**a**, **d** and **g**) compared to post-mortem Aβ antibody staining (**b**, **e** and **h**) in the merged images (**c**, **f** and **i**) revealed multiple dense cores within one larger plaque. *White arrows* indicate dense cores and *yellow arrowheads* new plaques in the vicinity (<40 μm) of pre-existing plaques. **j** Quantification of multicored plaques reveals that they become more numerous with increasing post-Methoxy-XO4 injection times. Mean ± SEM, *n* = 5–6 Kruskal–Wallis test with Dunn’s post hoc test *p < 0.05. **k** Positive correlation between the number of cores and plaque size 4 months after Methoxy-XO4 injection (linear regression *R* ^2^ = 0.07474; *p* < 0.0001, *n* = 687 plaques from 5 mice). *Scale bar* 25 μm

We observed that several new plaques can cluster in the vicinity of a single pre-existing plaque and characterized these clusters as ‘flower plaques’ due to their appearance of multiple new ‘petal’ plaques surrounding a central, Methoxy-XO4 positive plaque. We defined these flower plaques as two or more new plaques developing <40 μm away from the same pre-existing plaque. We identified these flower plaques with two-photon in vivo imaging after topical Aβ antibody administration (Fig. 3a–c) as well as with post-mortem antibody staining techniques (Fig. 3d–f). Of all ‘new in vicinity’ plaques, 10.5 % contributed to a flower plaque cluster (analysis of post-mortem images). The new plaques (‘petals’) can be of diffuse (Fig. 3b–c, e–f, yellow arrowheads) as well as of dense core nature (Fig. 3h–i, yellow arrowheads). To exclude the possibility that these ‘flower’ clusters were not simply an artifact caused by Methoxy-XO4, we stained age-matched naïve mice that did not receive Methoxy-XO4 prior to post-mortem staining and observed identical flower plaque structures (Suppl. Fig. 4a–c). To further investigate whether large plaques can form from multiple small precursor plaques, we analyzed the entire data set of plaques to find those which contained at least two distinct Methoxy-XO4 positive cores within an Aβ antibody positive plaque (≥2 separate Methoxy-XO4 positive particles). Examples of these multicore plaques are shown in Fig. 4a–i. We detected multicored plaques as early as 1 day post-Methoxy-XO4 injection (Fig. 4a–c) as well as in older animals (1 and 4 months post-Methoxy-XO4 injection, Fig. 4d–j). In all groups studied, 13 % of all large plaques (>300 μm^2^) were multicored, indicating that this population of plaques developed from more than one initial plaque or a cluster of plaques within the post-Methoxy-XO4 injection interval. Moreover, we found a highly significant positive correlation between the number of Methoxy-XO4 positive plaque cores contained within a plaque and the size of that plaque, showing that greater number of plaque cores corresponds to a larger plaque (Fig. 4k).

**Fig. 5:**
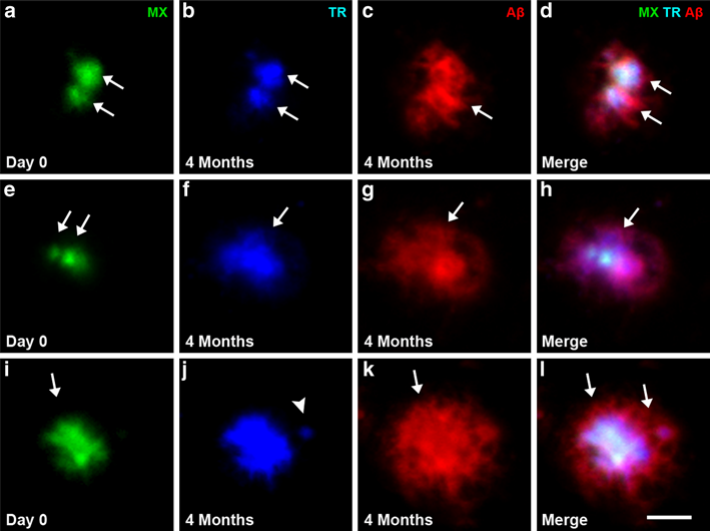
Fate of multicore plaques. Post-mortem Thiazin Red staining of dense core plaques (**b**, **f**, **j**) compared to Methoxy-XO4 pre-mortem staining 4 months prior to killing (**a**, **e**, **i**). Depicted are three different possibilities: **a**–**d** an example of non-merging multicores; **e**–**h** dense cores merging to a single dense core and **i**–**l** an example of a new dense core appearing within the diffuse halo around a pre-existing single dense core. *White arrows* point to multicores, the *white arrowhead* in **j** points to a newly developed dense core plaque. *Scale bar* 25 μm

**Fig. 6:**
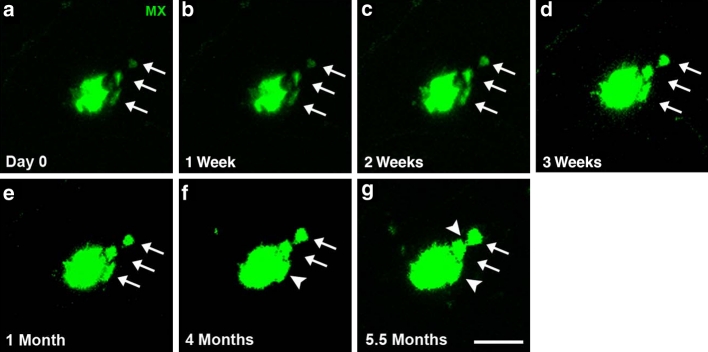
Cluster of plaques merging over time observed with in vivo imaging. Long-term, two-photon in vivo imaging shows an initial cluster of multiple, separate, dense core plaques (*white arrows*, **a**) that can be followed over subsequent weeks (**b**–**d**) to finally merge together after several months (*white arrowheads*, **e**–**g**). Methoxy-XO4 was injected 24 h before each imaging session. *Scale bar* 20 μm

Next, we addressed the question of whether multiple Methoxy-XO4 positive cores within one plaque (as defined by Aβ antibody staining) can fuse together over time, thereby giving rise to one large dense core plaque. To this end, we investigated the fate of multicore plaques after a 4 month post-Methoxy-XO4 injection period. We used Thiazin Red coupled with the Methoxy-XO4 and Aβ antibody staining to discern the nature of Aβ material. This triple labeling revealed that 75 % of multiple Methoxy-XO4 cores did not fuse together, but instead remained separate in the Thiazin Red staining (Fig. 5a–d). Notably, the remaining 25 % of multicored plaques did fuse together (Fig. 5e–h) demonstrating that dense cores can indeed merge over time and can give rise to larger dense core plaques. With long-term in vivo imaging over 5.5 months, we witnessed the fusion of dense core plaque clusters into a larger core, confirming our finding with a different technique (Fig. 6a–g). We also observed new dense cores emerging within the antibody halo of the Aβ antibody stain (Fig. 5i–l) indicating that multicore plaques can arise from plaques that develop sequentially.

**Fig. 7:**
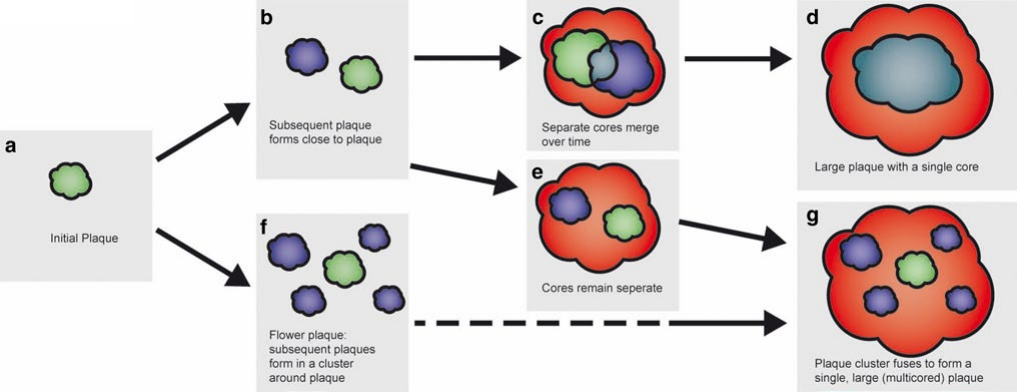
Schematic illustrating the clustering of amyloid plaque development. The initial plaque formation (**a**) can be followed by the formation of a new plaque close by **b**. These two plaques can merge over time (**c**) to form one large plaque with a single plaque core (**d**) or rather remain separate (**e**) as part of a larger plaque with multiple cores (**g**). Alternatively, the initial plaque (**a**) is followed by the clustering of new plaques around the initial plaque (**f**, flower plaque) and ultimately this cluster can merge together giving rise to one large plaque with multiple cores (**g**). *Solid arrows* refer to events captured by our data. The *dashed arrow* represents hypothetical plaque development

In summary, our data demonstrate that new plaques can deposit over time in the close vicinity of an initial plaque and that these multiple new plaques can cluster and give rise to the so-called ‘flower plaques’. Furthermore, large plaques can contain multiple pre-existing dense cores that can fuse over time supporting the novel hypothesis that multiple, individually small plaques cluster to ultimately build a larger plaque (Fig. 7).

## Discussion

In this study, we used pre- and post-mortem staining techniques to label plaques at multiple stages of plaque development. With this two-stage staining technique, we were able to visualize new plaques that had formed over time and reliably distinguish them from pre-existing plaques. Thereby, we unraveled the spatial relationship of newly deposited plaques to pre-existing plaques in detail. We made two important observations: (1) newly developed plaques are more likely to appear in the vicinity of pre-existing plaques than can be attributed to pure random deposition and (2) large plaques can be composed of multiple dense cores, indicating that these plaques are formed from initially small, individual plaques that clustered together and fused to become one larger plaque. Our data provide direct in vivo evidence that plaques can enlarge by forming clusters of multiple plaques, implying a mechanism whereby the development of large plaques is attributed in part to the clustering of pre-deposited and newly deposited plaques. This clustering concept unites the heretofore seemingly conflicting observations of little or no growth following the first appearance of plaques [6, 24], growth of plaques over extended periods of time [7, 13, 33] and an increasing size of plaques with age [27].

A first hint for plaque clustering came from computer simulations of plaque deposition, demonstrating that a mere random process could not account for the plaque deposition pattern observed in post-mortem brain sections. Rather, an influence of pre-existing plaques was necessary to successfully model the spatial pattern of plaque formation in AD pathology. Accordingly, simulated plaques formed preferentially in the vicinity of existing plaques [32]. Additional support for the notion that plaques can cluster comes from another study showing that plaques in APP transgenic mice are non-randomly distributed and more concentrated in certain areas of the barrel cortex [3], which led the authors to suggest that neuronal architecture could determine where plaques deposit. A similar study discovered a correlation between neuronal activity and the spatial deposition of plaques [4]. Together, these studies imply that plaques do not just randomly emerge in space, supporting our finding that newly developed plaques form in the vicinity of existing plaques at higher than chance levels.

The computer simulation of new plaque location we employed as a control analysis clearly showed the same effect—that new plaques formed in the vicinity of existing plaques at higher than chance levels. In older mice with higher plaque load, we observed the same trend. However, it did not reach statistical significance. We speculate that this is due to an underestimation of the number of ‘new in vicinity’ plaques in the older animals. There are three possible explanations to consider for this. First, the large, diffuse Aβ positive plaque ring-like structure that develops around the initial plaques could well obscure any very close newly appeared plaques. Second, we also observed clusters of plaques within the population of new plaques. These clusters of newly developed plaques suggest that once a new plaque has formed, subsequent new plaques cluster in the vicinity of this original new plaque. However, the temporal resolution of the method used here does not allow for this distinction. A third reason is the use of Methoxy-XO4. Although it has been shown that Methoxy-XO4 labels dense core plaques in vivo [6, 7, 13, 16, 17, 20, 31] and that it does not lose its staining capacity over long periods (up to 90 days) [9], we cannot rule out the possibility that it continues to label new plaques for some time after the initial application. Therefore, some plaques could be counted as pre-existing, although they appeared later and hence are actually new plaques. These three arguments support the notion that our method underestimates the amount of ‘new in vicinity’ plaques, implying that there may actually be many more newly emerged plaques in the vicinity of a pre-existing plaque. Another explanation for less close new plaques in animals with higher plaque density could be microglia that are recruited to the plaques [24] and take up Aβ material [6]. Therefore, new plaques would be less likely to form in the vicinity of pre-existing plaques in animals with a high plaque density and more peri-plaque microglia.

Our finding that plaques form preferentially in the vicinity of pre-existing plaques raises the question which mechanism might cause the formation of new plaques in the peri-plaque region. There is evidence that plaques can be induced by a seeding agent [22] and so it may be that pre-existing plaques produce seeds via diffusion or degradation processes, which induce the formation of neighboring plaques or represent a ‘pool’ of various Aβ conformations that further contribute to local toxicity and facilitate plaque formation [8, 11, 15, 18, 23]. One way in which plaques can exert this local toxicity is by the release of reactive oxygen species [21] resulting in oxidative stress on the surrounding cells which distorts neurites [10] and contributes to a decrease in membrane integrity in the vicinity of plaques [26]. Following initial plaque deposition, an immune response is triggered [24], which could be detrimental to the micro-environment [6] and increase the amount of plaque forming agents released from damaged neurons and glia. The micro-environment in which the initial plaque developed may be particularly favorable for additional plaque development [3, 4] so that multiple plaques preferentially develop within a very small area. Any or all of these factors could contribute to the non-random deposition of plaques in the vicinity of a pre-existing plaque.

Another important outcome of our study is the identification of multicored plaques. We observed many multicored plaques that clearly descended from more than one initial core. This emphasizes the clustering concept of large plaque formation since these plaques had to originate from a cluster of smaller precursors. We also found that the more cores a plaque has, the larger it tends to be, indicating that large plaques can be formed from many neighboring small plaques that developed over time. After 4 months post-injection, we observed different fates for these multicored plaques: they either stay separate but embedded within diffuse Αβ material or merge together to form a single large dense core. The idea of dense core plaque fusion is supported by previous work using a different mouse model of AD that reported the merger of several Methoxy-XO4 positive plaque cores over time [9]. Taken together, our results demonstrate the clustering of plaques and the subsequent merger of plaque clusters as a mechanism of large plaque formation.

Although the use of only one mouse model of AD is an important caveat to bear in mind, we believe that our results could pertain to a general mechanism of plaque growth. Our data suggest that a uniform growth of plaques from a single seed may provide only a partial explanation of how large plaques develop over time and rather complements a uniform growth model of plaque development by adding a new clustering dimension. We propose a multi-faceted clustering mechanism whereby new plaques can deposit close to existing plaques forming a cluster of small plaques. This cluster can then ultimately merge over time to give rise to the single, large plaques that we observe in aged mice and autopsy material of AD patients. The clustering hypothesis as a mechanism of plaque formation and growth suggests that the micro-environment becomes conducive to plaque deposition. Whether this is the first plaque leading to a greater chance of another plaque forming nearby or a reflection of something unique in that microenvironment that leads to both the first deposit and the increased chance of subsequent deposits remain uncertain. In either case, these observations of plaque development deepen our understanding of plaque formation and plaque growth in Alzheimer’s disease.

### **Author contributions**

J.F.M. performed all the experiments and data analyses (apart from chronic in vivo imaging and computer simulations). S.L. and M.H. carried out chronic in vivo two-photon imaging experiments and performed the computer simulation analysis. C.A.A. provide technical assistance. M.M.-L. together with B.T.H. initiated the project. J.F.M., S.L., T.B., B.T.H., C.H. and M.M.-L. discussed the results. J.F.M. and M.M.-L. wrote the manuscript and M.M.-L. coordinated the study. All authors edited the paper.

## Electronic supplementary material

Below is the link to the electronic supplementary material.

Supplementary Figure 1 New dense core plaques. Premortem Methoxy-XO4 staining as shown in green (a) compared to subsequent postmortem Methoxy-XO4 staining and imaging as shown in red (b) reveals new Methoxy-XO4 positive plaques (white arrowheads, c). Postmortem Thiazin Red staining (e in blue) shows a new dense core plaque (white arrowhead, f) 4 months after Methoxy-XO4 injection (d) (JPEG 385 kb)

Supplementary Figure 2 Diffuse Aβ detected with 4G8 antibody. Immunhistochemical stainings of plaques with 4G8 antibody 1 day (a-d), 1 month (e–h) and 4 months (i-l) after Methoxy-XO4 injection revealed increasing amounts of diffuse amyloid surrounding the dense core plaque with time. Scale bar: 25 μm (JPEG 661 kb)

Supplementary Figure 3 ‘New in the vicinity’ plaques compared to simulated new plaques. Computer-simulated plaque analysis revealed that the proportion of new plaques originally in the vicinity of pre-existing plaques was significantly higher than simulated new plaques in the young post injection group (a). This difference did not reach statistical significance in the 1 month and 4 months post injection groups (b). One data point represents one animal. Mean ± SEM, n = 5-9 per group; Wilcoxon matched-pairs signed rank test* p < 0.05, n.s. p > 0.05. The number of plaques per ROI increases with incubation time (c). Mean ± SEM, n = 5-6 per group, each symbol represents one animal (JPEG 374 kb)

Supplementary Figure 4 Postmortem Methoxy-XO4 staining. Examples of brain sections stained postmortem with Methoxy-XO4 of an age matched naïve mouse (a-c). (a) A ‘flower plaque’ consisting of small plaques (white arrowheads) clustering around a larger plaque, (b) clusters of similar sized plaques and (c) seemingly fused plaque clusters. Scale bar: 25 μm (JPEG 382 kb)

Supplementary Table 1 Table displaying the number of animals used in each experimental group (DOCX 15 kb)
