# Supplementary figures and images for: Clustering of plaques contributes to plaque growth in a mouse model of Alzheimer’s disease
Source: Acta Neuropathol. 2013 Jun 18;126(2):179–88. doi: 10.1007/s00401-013-1137-2 (PMC3722456; doi:10.1007/s00401-013-1137-2)

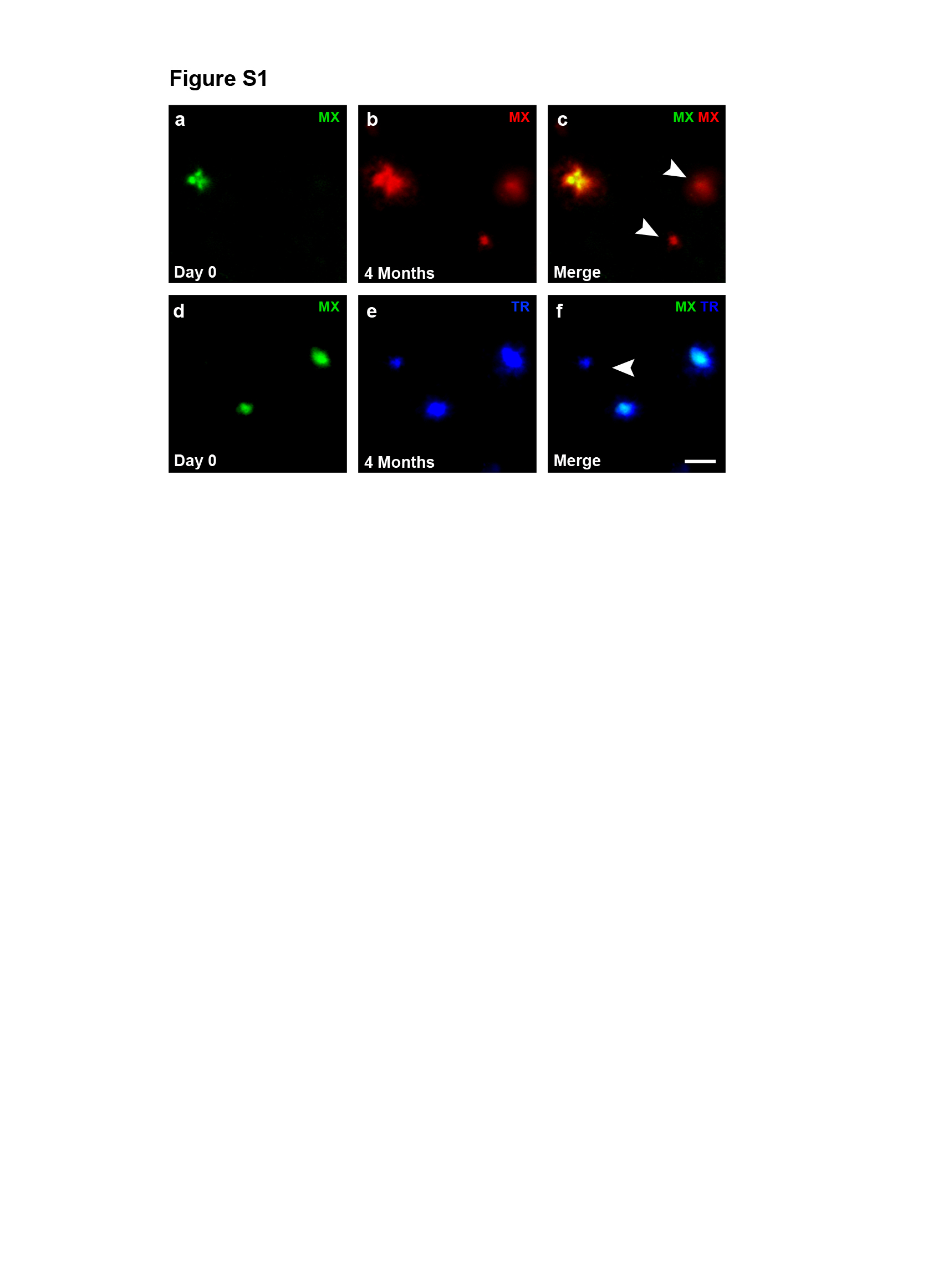

Supplement: Supplementary file 1 — Supplementary Figure 1 New dense core plaques. Premortem Methoxy-XO4 staining as shown in green (a) compared to subsequent postmortem Methoxy-XO4 staining and imaging as shown in red (b) reveals new Methoxy-XO4 positive plaques (white arrowheads, c). Postmortem Thiazin Red staining (e in blue) shows a new dense core plaque (white arrowhead, f) 4 months after Methoxy-XO4 injection (d) (JPEG 385 kb) [file 401_2013_1137_MOESM1_ESM.jpg]

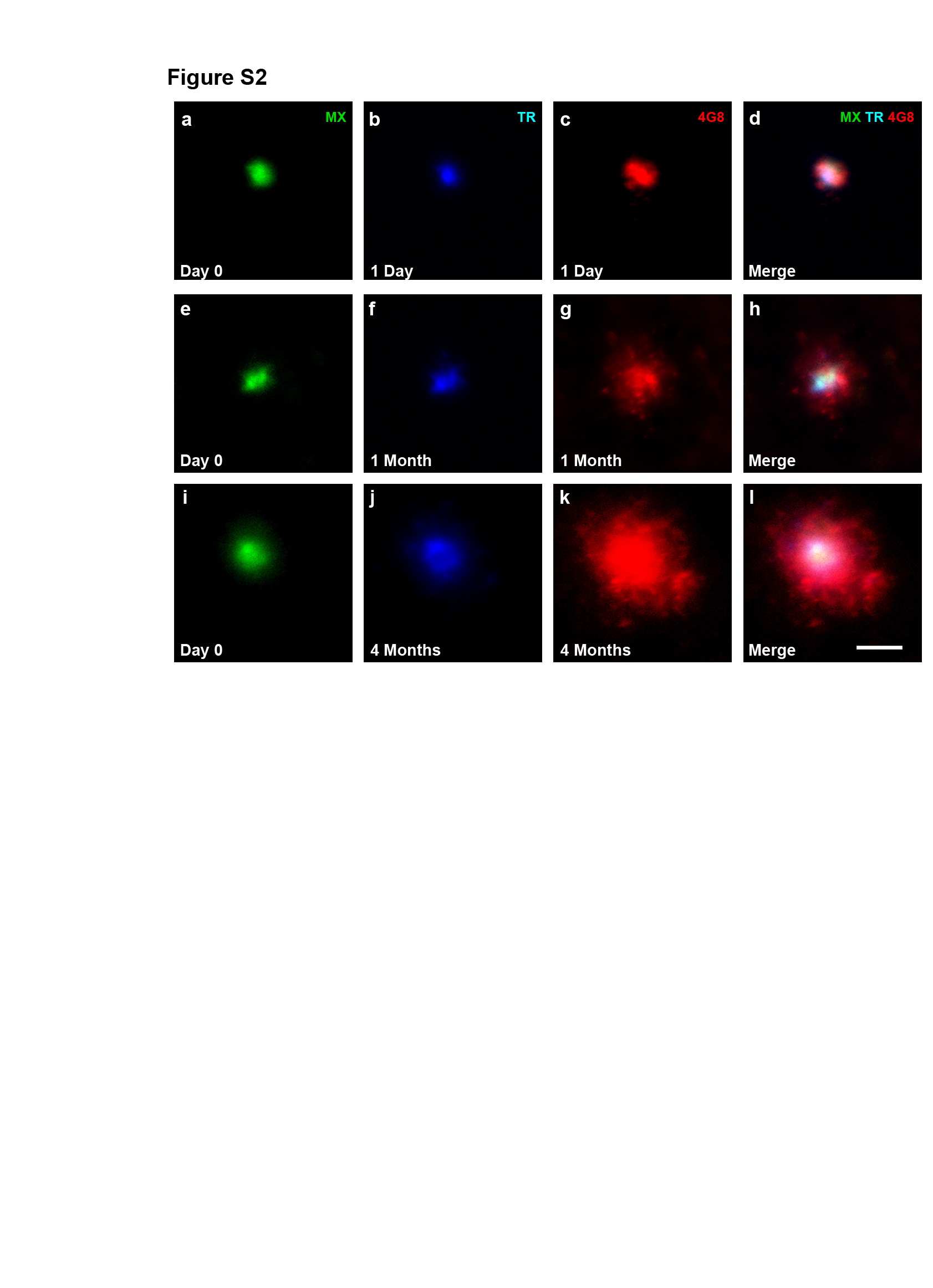

Supplement: Supplementary file 2 — Supplementary Figure 2 Diffuse Aβ detected with 4G8 antibody. Immunhistochemical stainings of plaques with 4G8 antibody 1 day (a-d), 1 month (e–h) and 4 months (i-l) after Methoxy-XO4 injection revealed increasing amounts of diffuse amyloid surrounding the dense core plaque with time. Scale bar: 25 μm (JPEG 661 kb) [file 401_2013_1137_MOESM2_ESM.jpg]

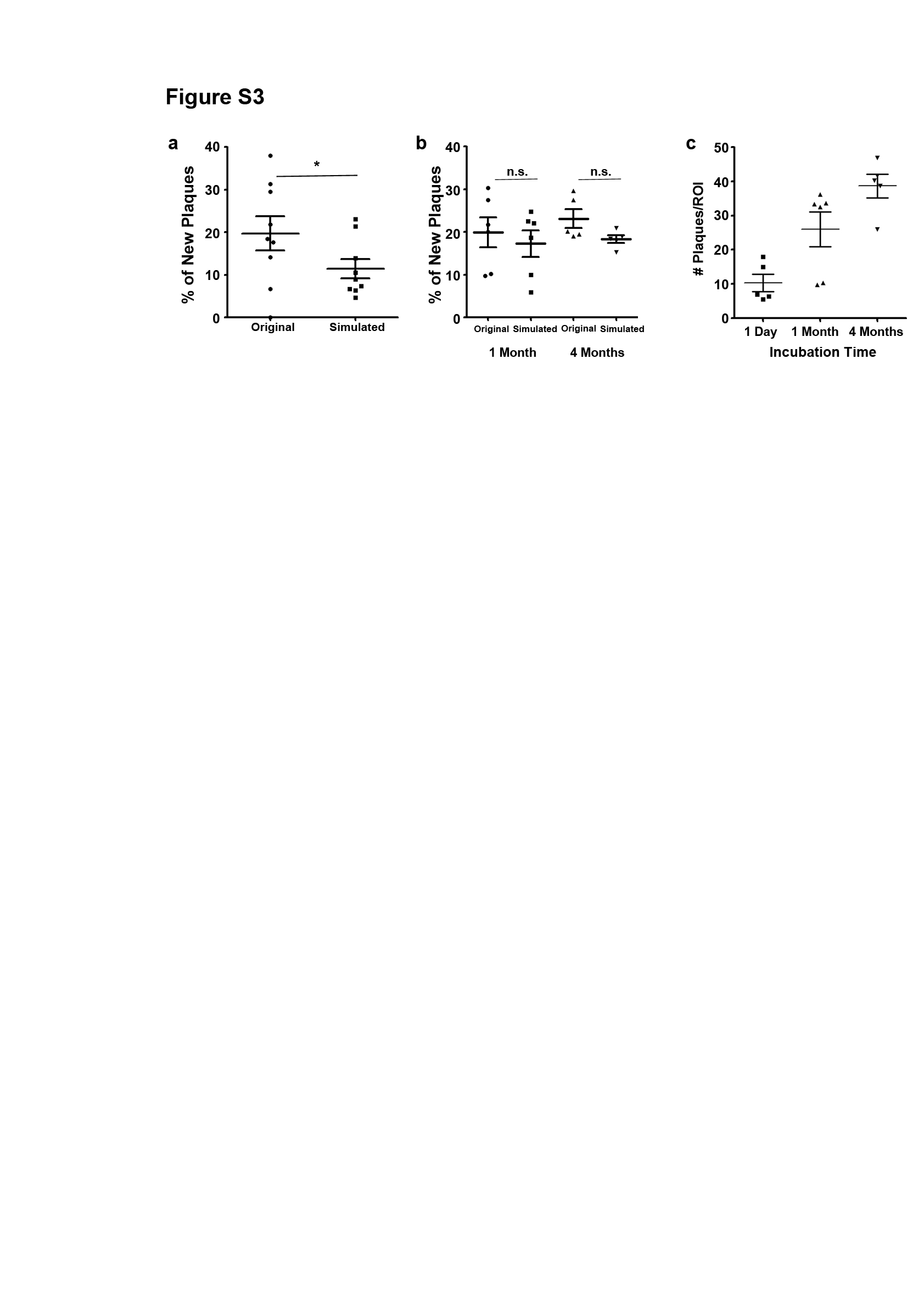

Supplement: Supplementary file 3 — Supplementary Figure 3 ‘New in the vicinity’ plaques compared to simulated new plaques. Computer-simulated plaque analysis revealed that the proportion of new plaques originally in the vicinity of pre-existing plaques was significantly higher than simulated new plaques in the young post injection group (a). This difference did not reach statistical significance in the 1 month and 4 months post injection groups (b). One data point represents one animal. Mean ± SEM, n = 5-9 per group; Wilcoxon matched-pairs signed rank test* p < 0.05, n.s. p > 0.05. The number of plaques per ROI increases with incubation time (c). Mean ± SEM, n = 5-6 per group, each symbol represents one animal (JPEG 374 kb) [file 401_2013_1137_MOESM3_ESM.jpg]

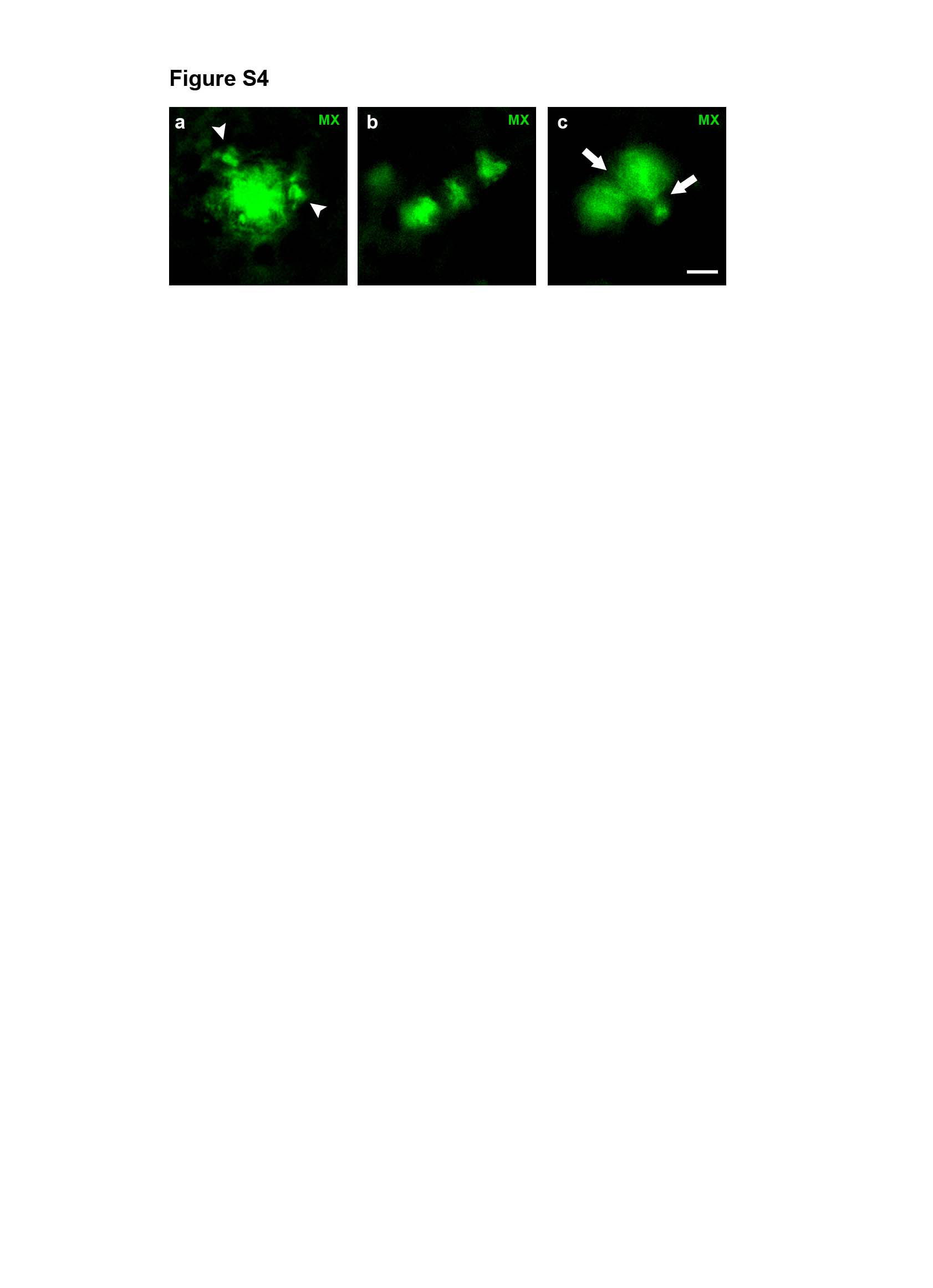

Supplement: Supplementary file 4 — Supplementary Figure 4 Postmortem Methoxy-XO4 staining. Examples of brain sections stained postmortem with Methoxy-XO4 of an age matched naïve mouse (a-c). (a) A ‘flower plaque’ consisting of small plaques (white arrowheads) clustering around a larger plaque, (b) clusters of similar sized plaques and (c) seemingly fused plaque clusters. Scale bar: 25 μm (JPEG 382 kb) [file 401_2013_1137_MOESM4_ESM.jpg]
