# Supplementary material for: Clustering of plaques contributes to plaque growth in a mouse model of Alzheimer’s disease
Source: Acta Neuropathol. 2013 Jun 18;126(2):179–88. doi: 10.1007/s00401-013-1137-2 (PMC3722456; doi:10.1007/s00401-013-1137-2)
Supplement: Supplementary file 5 — Supplementary Table 1 Table displaying the number of animals used in each experimental group (DOCX 15 kb) [file 401_2013_1137_MOESM5_ESM.docx]

**Supplementary Table 1** *Table showing the number of animals used in each experimental group.*

| Group name | Animal age at MX injection | Post injection interval | Number of animals |
| --- | --- | --- | --- |
|  | 2 months | 2 weeks | 4 |
| 1 Day | 3 months | 1 day | 5 |
| 1 Month | 3 months | 1 month | 6 |
| 4 Months | 3 months | 4 months | 5 |
| Acute *in vivo* imaging | 3 months | 4 months | 3 |
